# Therapeutic Efficacy of IL7/CCL19-Expressing CAR-T Cells in Intractable Solid Tumor Models of Glioblastoma and Pancreatic Cancer

**DOI:** 10.1158/2767-9764.CRC-24-0226

**Published:** 2024-09-25

**Authors:** Keisuke Ohta, Yukimi Sakoda, Keishi Adachi, Taro Shinozaki, Masao Nakajima, Hiroyuki Yasuda, Hiroaki Nagano, Koji Tamada

**Affiliations:** 1 Department of ImmunologyYamaguchi University Graduate School of Medicine, Ube, Japan.; 2 Department of Gastroenterological, Breast and Endocrine Surgery, Yamaguchi University Graduate School of Medicine, Ube, Japan.; 3 Department of Pulmonary Medicine, Keio University School of Medicine, Shinjuku-ku, Tokyo.

## Abstract

Cancer immunotherapy using immune checkpoint inhibitors and its combination with other anticancer therapies has emerged as a new standard of care because of the encouraging therapeutic effects in various solid cancers. Nonetheless, glioblastoma and pancreatic cancer remain resistant to immunotherapy and represent intractable cancers with the poorest prognosis. We investigated the therapeutic effects of next-generation chimeric antigen receptor (CAR) T cells producing IL7 and chemokine (C–C motif) ligand 19 (CCL19; referred to as 7 × 19 CAR-T) in these intractable cancers. Cytotoxic activities and therapeutic effects of 7 × 19 CAR-T were evaluated *in vitro* and *in vivo*, in a model using EGFR variant III (EGFRvIII)–positive glioblastoma and anti-EGFRvIII CAR-T generated from healthy donor peripheral blood mononuclear cells (PBMC), or a model using HER2-positive pancreatic cancer organoids and anti-HER2 CAR-T generated from the same patient’s PBMC. Anti-EGFRvIII 7 × 19 CAR-T exhibited cytotoxic activity specific to EGFRvIII-positive tumor, induced complete rejection of glioblastoma with massive T-cell infiltration and tumor cell death in the tumor tissues, and consequently prolonged mouse survival. Anti-HER2 7 × 19 CAR-T demonstrated a potent cytotoxic activity against autologous HER2-positive pancreatic cancer organoids and induced complete rejection of autologous tumor along with prolonged mouse survival. Our results suggest that 7 × 19 CAR-T could become a therapeutic option for glioblastoma and pancreatic cancer. To the best of our knowledge, this is the first study to demonstrate the therapeutic efficacy of next-generation CAR-T in an autologous model using patient-derived tumor organoids and CAR-T generated from the same patient’s PBMC, in which unwanted allogeneic immune responses are fully excluded.

**Significance:**

Despite the clinical development of CAR T-cell therapy, its efficacy in solid cancers has yet to be established. This study explored the therapeutic potential and immunologic mechanisms of IL7/CCL19-producing CAR-T therapy in preclinical solid cancer models of glioblastoma and pancreatic cancer. We found that IL7/CCL19-producing CAR-T cells generated from the patient’s PBMC showed potent therapeutic effects against the solid cancer model established by inoculating organoids from the autologous tumor tissue.

## Introduction

The overall survival (OS) of various malignant tumors has been improved by recent advances in cancer immunotherapy using immune checkpoint inhibitors (ICI) and its combination with other anticancer drugs, which are currently approved as standardized treatments for patients with advanced cancer (1, 2). Nonetheless, glioblastoma and pancreatic cancer still represent the intractable solid cancers with the poorest prognosis due to no effective immunotherapies. Subsequent to ICI, chimeric antigen receptor (CAR) T therapy emerged as a novel and promising immunotherapy, as it has demonstrated significant efficacy in patients with advanced B-cell malignancies and multiple myeloma (3–8). However, the current approaches of CAR-T therapies remain ineffective for glioblastoma, pancreatic cancers, and other solid cancers (9, 10).

Glioblastoma accounts for about half of the central nervous system tumors in adults, showing very poor prognosis with only 5% in a 5-year survival rate by the standard of care (11, 12). CAR-T therapies targeting HER2 or EGFR variant III (EGFRvIII) have been investigated for the treatment of glioblastoma (13–16). In the phase I trial of anti-HER2 CAR-T with 16 patients, 1 had a partial response for more than 9 months, 7 had a stable disease for 8 weeks to 29 months, and 8 progressed after T-cell infusion, resulting in a median OS of 11.1 months (95% confidence interval, 4.1–27.2 months; ref. 13). In the phase I trial of anti-EGFRvIII CAR-T, 1 of 18 patients survived for 59 months, whereas the others showed no tumor shrinkage (15). The median progression-free survival (PFS) and OS were 1.3 and 6.9 months, respectively. Overall, the efficacy of the current CAR-T therapies for glioblastoma is limited and insufficient.

In pancreatic cancer, the 5-year survival rate by the current standard therapies is approximately 10% (17), and clinical trials of CAR-T resulted in insufficient efficacy so far. In the phase I study of anti-HER2 CAR-T in 11 patients with pancreatic cancer, 1 showed partial response whereas 5 showed stable disease, and the median PFS was 4.8 months (18). In the phase I study of CAR-T targeting mesothelin, only two of six patients showed stable disease with a PFS of 3.8 and 5.4 months (19). Taken together, previous attempts of CAR-T therapies in glioblastoma and pancreatic cancer resulted in poor outcomes in efficacy, although no severe safety concerns have been reported. Thus, novel technologies to make CAR-T more offensive to these intractable tumors are of both urgent and great demand.

Our group has developed the novel CAR-T which simultaneously produces IL7 and chemokine (C–C motif) ligand 19 (CCL19; referred to as 7 × 19 CAR‐T; refs. 20–22). 7 × 19 CAR‐T induces active migration of T cells and dendritic cells through the effect of CCL19 and promotes T-cell proliferation and memory formation through the effect of IL7. Administration of 7 × 19 CAR-T induced potent therapeutic effects in various solid tumor models using syngenetic mouse tumors and patient-derived xenograft tumors, in which the conventional CAR-T cells (referred to as Conv. CAR-T) showed no or little effects. We reported that 7 × 19 CAR-T mobilizes endogenous T cells to the tumor site and converts cold tumors into hot tumors and that the endogenous T cells are responsible, at least in part, for the antitumor effects of the treatment, suggesting a unique mechanism different from other armored CAR-T technologies. The goal of this study is to apply the IL7/CCL19‐producing technology to make anti-EGFRvIII or HER2 CAR-T more offensive to glioblastoma and pancreatic cancer, so as to improve the therapeutic efficacy.

In most experimental models using human solid tumors, CAR-T is generated from the healthy donor peripheral blood mononuclear cells (PBMC), in which MHC is not identical between tumors and CAR-T. The mismatch of MHC induces allogeneic T-cell responses which could modify antitumor efficacy, leading to inaccurate assessment of CAR-T functions and efficacy. To avoid such unwanted influences, experiments should be performed in a model with autologous transplantation, in which tumors and CAR-T are derived from the same patient origin. In addition, T cells in patients with cancer are largely dysfunctional (23), and the T-cell number and ratios of the naïve/stem cell memory (SCM) phenotypes in patients with multiple myeloma are low compared with those of healthy donors (24), suggesting functional differences in CAR-T generated from patients with cancer or healthy donors. In this context, it is also important to perform experiments by using CAR-T generated from PBMC of patients with cancer. Therefore, this study established an autologous tumor model in which the immunodeficient mice inoculated with patient-derived tumor organoids were treated with CAR-T generated from the same patient’s PBMC. To the best of our knowledge, this is the first study to investigate the efficacy of CAR-T in the autologous model of solid cancers.

## Materials and Methods

### Mice and cell lines

Female NOD.Cg-*Prkdc*^*scid*^*Il2rg*^*tm1Sug*^*B2m*^*em1Tac*^*H2-Ab1*^*tm1Doi*^/Jic (NOG-ΔMHC) mice aged 7 to 8 weeks were purchased from CLEA Japan and used for *in vivo* experiments. The mice were maintained under specific pathogen-free conditions in our facility as previously reported (21, 22) and given enrofloxacin orally for a week after arrival or until the end of experiments. All animal experiments were approved by the Institutional Animal Care and Use Committee in Yamaguchi University. U87MG (RRID: CVCL_0022), a human glioblastoma cell line, was purchased from ATCC. U87MG was genetically modified to stably express EGFRvIII, and EGFRvIII-positive U87MG was established by cell sorting with SH800 (Sony) at Yamaguchi University (referred to as U87MG EGFRvIII). U87MG and U87MG EGFRvIII cells were cultured in minimum essential medium (Gibco) supplemented with 10% heat-inactivated FBS (CCP) and 1% penicillin–streptomycin sulfate (Wako). These cell lines were confirmed negative for *Mycoplasma* testing and used for the experiments within five passages in this study.

### Design of CAR vectors and gene transfer to human T cells

A hybridoma-producing anti-EGFRvIII mAb was generated by immunizing mice with EGFRvIII peptide (Cell Engineering Corporation). Anti–EGFRvIII single-chain variable fragment (scFv) was composed of variable region sequences of heavy and light chains of hybridoma. CAR construct was designed with the anti–EGFRvIII scFv, the transmembrane sequence of human CD8α, and cytoplasmic sequences of human 4-1BB and CD3ζ and cloned into the retroviral pMSGV1 vector (25). Anti–HER2 human scFv was designed with variable region sequences of heavy and light chains of trastuzumab. A CAR construct was designed with the anti-HER2 scFv, the transmembrane sequence of human CD8α, and cytoplasmic sequences of human 4-1BB, CD28 and CD3ζ and cloned into the retroviral pMSGV1 vector. IL7, CCL19, and HSV-TK were expressed in addition to anti-EGFRvIII or anti-HER2 CARs by connecting the self-cleavable 2A liner sequence. Gene transfer to human T cells was conducted as previously described (21, 22, 25, 26). Briefly, retroviral vectors were produced by the transfection of CAR-expressing plasmids into GP2-293 packaging cells together with the pAmpho envelope vector plasmid (Retro-X Universal Packaging System, Clontech). The culture supernatants containing the retroviral vector were harvested and used for infection to activate PBMC derived from healthy donors or patients with cancer, in the presence of RetroNectin reagent (Takara Bio) and anti-CD3 mAb (clone OKT3, eBioscience). The cells were cultured with OpTmizer (Gibco) supplemented with OpTmizer CTS, CTS Immune Cell serum replacement, L-Glutamine (Gibco), 1% penicillin–streptomycin sulfate, and amphotericin B (Bristol Myers Squibb) in the presence of IL2. In some experiments, retroviral vectors were harvested from the producer cell line which was generated by stable transduction of the CAR-expressing gene together with the envelope gene into the packaging cell line. The transduction efficiency of CAR was assessed by flow cytometry. The production of human IL7 and CCL19 in the culture supernatants was measured as previously reported (21, 22). Untransduced (UTD) activated T cells, which were used as controls, were generated under the same culture conditions without retrovirus infection.

### Flow cytometry

For the detection of EGFRvIII, unconjugated anti-EGFRvIII mAb, which was obtained from the supernatant of the anti-EGFRvIII hybridoma (Cell Engineering Corporation), and secondary APC-conjugated anti–mouse IgG Ab (clone poly4053, BioLegend) were used. Unconjugated trastuzumab (Chugai Pharmaceutical Co., Ltd) and secondary PE-conjugated anti–human IgG Ab (Thermo Fisher Scientific) were used to detect HER2. The CAR-expressing T cells were detected by APC-conjugated anti–human CD8 mAb (clone HIT8a, BioLegend) together with either biotinylated anti-CAR linker Ab (Cell Engineering Corporation) followed by PE-conjugated streptavidin (BioLegend) for anti-EGFRvIII CAR or human recombinant ErbB2/Her2-Fc protein (R&D Systems) followed by PE-conjugated anti–human IgG Ab (Thermo Fisher Scientific) for anti-HER2 CAR. CAR-T cells were further stained to determine the immunophenotype with APC/Cy7-conjugated anti–human CD3 mAb (Clone HIT3a, BioLegend), PerCP/Cy5.5-conjugated anti–human CD4 mAb (Clone OKT4, BioLegend), FITC-conjugated anti–human CD45RA mAb (clone HI100, BioLegend), and BV421-conjugated anti–human CCR7 mAb (clone 150503, BD Biosciences). Zombie Yellow viability dye (BioLegend) and APC/Cy7-conjugated anti–human CD3 mAb (clone HIT3a, BioLegend) were used to evaluate viable immune and nonimmune cells after *in vitro* coculture assay. Human TruStain FcX (BioLegend) was used to block nonspecific binding of mAb with Fcγ receptors. Flow cytometric data were acquired using CytoFLEX (Beckman Coulter), and the obtained data were analyzed using FlowJo software (RRID: SCR_008520; FlowJo, LLC).

### *In vitro* cytotoxicity assay against U87MG EGFRvIII

To assess the cytotoxic activity *in vitro*, anti-EGFRvIII CAR-T or UTD T cells were cocultured with EGFRvIII-positive or -negative tumor cells at various ratios for 2 days. The number of remaining tumor cells and the production of IFNγ in the supernatant were examined as previously reported (21, 22). The percentage of CAR-positive cells, which varied among the CAR constructs, was adjusted to the same level by adding UTD T cells prior to the coculture.

### *In vivo* antitumor model against U87MG EGFRvIII

NOG-ΔMHC mice were inoculated subcutaneously with 3.5 × 10^5^ U87MG EGFRvIII tumor cells on the right flank on day 0. Ten days later, the mice were randomized, and 3 × 10^6^ anti-EGFRvIII CAR-T or 1×10^7^ UTD T cells were injected intravenously through the tail vein. The percentage of CAR-positive cells, which varied among the CAR constructs, was adjusted to the same level by adding UTD T cells prior to the injection. Tumor size was assessed as previously reported (21, 22), and the mice were euthanized when the tumor diameter exceeded 20 mm. Mice suffering from progressive GVHD with body weight loss were excluded from the analysis. In this experiment, PBMC from a single healthy donor were used for the generation of CAR-T or UTD T cells.

To analyze T-cell infiltration in the tumor tissues, the tumor mass was resected 3 days after intravenous administration of CAR-T and fixed with 10% formaldehyde followed by embedding in paraffin. The tissue sections were analyzed by hematoxylin and eosin (H&E) staining or IHC staining with rabbit anti-CD4 mAb (clone SP35, Roche), rabbit anti-CD8 mAb (clone SP57, Roche), rabbit anti-CCR7 mAb (clone EPR23192-57, Abcam), rabbit anti–caspase-3 mAb (clone Asp175, Cell Signaling Technology), and mouse anti-EGFRvIII mAb (Cell Engineering Corporation). Microscopic analyses for H&E and IHC samples were conducted using a BZ-X710 fluorescence microscope and BZ-X analyzer (KEYENCE).

For T-cell receptor (TCR) repertoire analysis, T cells were harvested from the mouse spleen on day 42 after CAR-T administration and sorted into CAR-positive and -negative cells. The sorted T cells were dissolved in ISOGEN-LS (NIPPON GENE) and cryopreserved. Purification of RNA and analysis with the next-generation sequencer were conducted by Repertoire Genesis.

### Establishment of human pancreatic cancer organoids

Clinical samples used for organoid establishment were obtained from patients at Yamaguchi University Hospital with written informed consent from the patients. The studies were conducted in accordance with the Declaration of Helsinki and with the approval of the Institutional Review Board of Yamaguchi University. The specimens of pancreatic ductal adenocarcinoma were collected along with surgical resection and used for establishing organoids as previously reported with some modifications (27, 28). First, surgical specimens were washed vigorously and minced into fragments of 10 mm^3^ size using surgical scissors. The fragments were digested with Liberase TH (Roche) at 37°C for 20 minutes and incubated with Red Blood Cell Lysis Buffer (Roche) to eliminate red blood cells. The digested cells were washed with PBS containing 10% FBS to inactivate the digestive enzyme, followed by incubation. The obtained pancreatic cancer cells were embedded in Matrigel (Corning) and overlaid with a basal culture medium (27, 28), namely, advanced DMEM/F12 supplemented with penicillin/streptomycin, 10 mmol/L HEPES, 2 mmol/L GlutaMAX, 1 × B27 (Thermo Fisher Scientific), 10 nmol/L gastrin I (Sigma), 1 mmol/L N-acetylcysteine (Sigma), 50 ng/mL mouse recombinant EGF (Thermo Fisher Scientific), 50 ng/mL human recombinant FGF2 (PeproTech), 100 ng/mL human recombinant IGF1 (BioLegend), 100 ng/mL mouse recombinant noggin (PeproTech), 10% R-spondin-1 (R&D Systems), 25% Afamin/Wnt3a serum-free conditioned medium (MBL), 500 nmol/L A83-01 (R&D Systems), and 10 µmol/L Y-27632 (Nacalai Tesque). To enrich pancreatic cancer–derived organoids, EGF, FGF2, and IGF1 were removed from the medium or 3 µmol/L nutlin 3 (Cayman Chemical) was added to the medium. The established organoids were cryopreserved as previously described (27, 28).

### *In vitro* cytotoxicity assay against HER2-positive pancreatic cancer organoid cells

To assess a cytotoxic activity *in vitro*, anti-HER2 CAR-T and UTD T cells generated from PBMC of patients with pancreatic cancer were cocultured with pancreatic cancer organoids established from the same patient for 4 days. The number of remaining tumor cells and the production of IFNγ in the supernatant were examined as previously reported (21, 22). The percentage of CAR-positive cells, which varied among the CAR constructs, was adjusted to the same level by adding UTD T cells prior to the coculture.

### *In vivo* antitumor mouse model using HER2-positive pancreatic cancer organoids

For the *in vivo* model, NOG-ΔMHC mice were inoculated subcutaneously in the right flank with 1 × 10^6^ pancreatic cancer organoid cells in 100 μL solution composed of 50:50 Matrigel (Corning) and Hank’s Balanced Salt Solution (Thermo Fisher Scientific) on day 0. The mice were treated with intravenous injection of 5 × 10^6^ CAR-T generated from the same patient’s PMBC on day 10 or left untreated. The percentage of CAR-positive cells, which varied among the CAR constructs, was adjusted to the same level by adding UTD T cells prior to the injection. Tumor size was assessed as previously reported (21, 22), and the mice were euthanized when the tumor diameter exceeded 15 mm pancreatic cancer organoid. In this experiment, PBMC from a single healthy donor were used for the generation of CAR-T or UTD T cells.

To analyze HER2 expression in the transplanted organoids, tumor mass was resected from the mice, fixed with 10% formaldehyde, and then embedded in paraffin. The tissue sections were analyzed by IHC staining with rabbit anti-HER2 mAb (clone 4B5, Roche). The original tumor specimens obtained by surgical resection and used for organoid establishment were also analyzed for HER2 expression by IHC staining. Microscopic analyses for IHC samples were conducted using a BZ-X710 fluorescence microscope and BZ-X analyzer.

### Statistics

Statistical analyses of *in vitro* experiments and *in vivo* mouse survival assays were examined using the two-sided Student *t* test and log-rank test, respectively. *P* < 0.05 was considered statistically significant.

### Data availability

The authors confirm that the data supporting the findings of this study are available within the article and its Supplementary Materials. The data generated in this study are available upon request from the corresponding author.

## Results

### Generation and functional analyses of anti-EGFRvIII CAR-T expressing IL7 and CCL19

Our previous studies have revealed a significant enhancement in the therapeutic efficacy of 7 × 19 CAR-T in several solid tumor models (20–22). To apply the therapeutic efficacy of 7 × 19 CAR-T to EGFRvIII-positive glioblastoma, a second-generation CAR construct containing anti–EGFRvIII scFv, hinge and transmembrane region, 4-1BB, and CD3ζ signaling motifs, together with coexpression of IL7 and CCL19 sequences was designed (Supplementary Fig. S1A). As a control, Conv. CAR without IL7 and CCL19 expression sequences was also constructed. When human PBMC were transduced with the retroviral vector of either Conv. CAR or 7 × 19 CAR, the transduction efficiencies of CAR were 56.8% and 63.6%, respectively (Supplementary Fig. S1B). UTD T cells were used as non-CAR control T cells. Human IL7 and CCL19 were abundantly detected in the culture supernatant of 7 × 19 CAR-T, but neither in those of Conv. CAR-T and UTD (Supplementary Fig. S1C). These results confirmed that anti-EGFRvIII 7 × 19 CAR-T was successfully generated.

**Figure 1:**
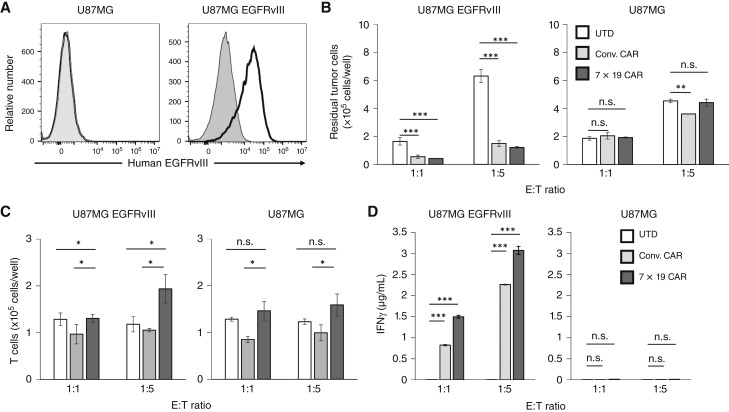
EGFRvIII-specific immune responses of Conv. CAR and 7 × 19 CAR-T cells *in vitro*. **A,** Surface expression of EGFRvIII was assessed in U87MG glioblastoma cell lines by flow cytometry. Open and filled histograms indicate staining with anti-EGFRvIII Ab and nonstaining, followed by anti–mouse IgG Ab, respectively. **B** and **C,** Conv. CAR-T, 7 × 19 CAR-T, or UTD T cells were cocultured with the indicated tumor cells at an effector-to-target (E:T) ratio of 1:1 and 1:5 for 2 days. The number of residual tumor cells (**B**) and T cells (**C**) after coculture was analyzed by flow cytometry. Data are shown as mean ± SD of triplicate samples. **D,** The supernatants of the coculture described in **B** were harvested, and the concentration of IFNγ was assessed by ELISA. Data are shown as mean ± SD of triplicate samples. **, *P* < 0.005; ***, *P* < 0.001; n.s., not significant.

We next evaluated the functions of anti-EGFRvIII 7 × 19 CAR-T *in vitro* by coculture with U87MG and U87MG EGFRvIII glioblastoma cell lines (Fig. 1A). The residual cell number of U87MG EGFRvIII tumors was significantly reduced after coculture with Conv. or 7 × 19 CAR-T, but not UTD, whereas the reduction of tumor cells was undetected or less when EGFRvIII-negative U87MG tumors were used for the coculture (Fig. 1B), indicating EGFRvIII-specific tumor lysis by CAR-T. After coculture, the cell number of 7 × 19 CAR-T was significantly higher than that of Conv. CAR-T (Fig. 1C), suggesting a role of IL7 in promoting the proliferation and survival of CAR-T, consistent with our previous report (20). In addition, IFNγ production by Conv. and 7 × 19 CAR-T, but not UTD, was observed in the coculture with U87MG EGFRvIII tumors, whereas the level of IFNγ production by 7 × 19 CAR-T was relatively higher than that by Conv. CAR-T (Fig. 1D). When Conv. or 7 × 19 CAR-T cells were cocultured with U87MG tumor, negligible levels of IFNγ were detected. These results confirmed the EGFRvIII antigen–specific immune responses of anti-EGFRvIII 7 × 19 CAR-T (Fig. 1).

### Potent therapeutic effects of anti-EGFRvIII 7 × 19 CAR-T in a solid tumor model of human glioblastoma

**Figure 2:**
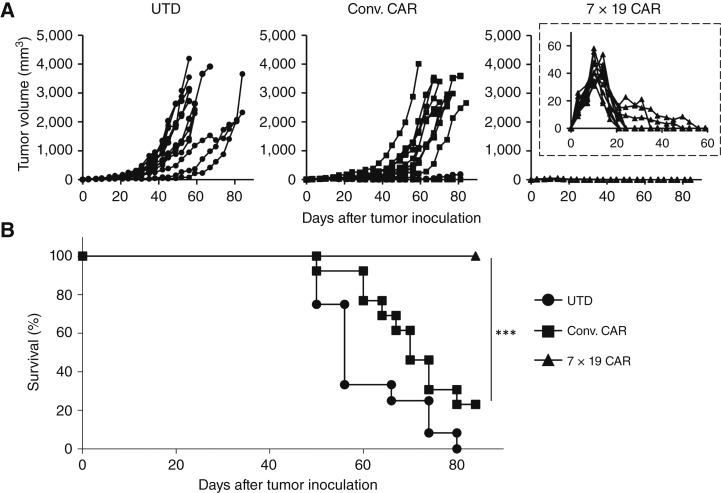
Potent therapeutic effects of 7 × 19 CAR-T in the preestablished solid tumor model of human glioblastoma. NOG-ΔMHC mice were inoculated subcutaneously with 3.5 × 10^6^ U87MG EGFRvIII tumor cells on day 0 and then treated with intravenous injection of 1 × 10^7^ Conv. CAR-T, 7 × 19 CAR-T, or UTD on day 10. Thereafter, the tumor size (**A**) and the mouse survival (**B**) were assessed (*n* = 12 each in UTD and 7 × 19 CAR-T groups; *n* = 13 in Conv. CAR-T group; *n* values are biological replicates). In **A**, each line indicates the tumor volume of an individual mouse. Tumor volumes during the initial 56 days of 7 × 19 CAR-T–treated mice are shown in the inset. ***, *P* < 0.001.

To investigate the therapeutic effects of anti-EGFRvIII CAR-T *in vivo*, we developed a preestablished solid tumor model of human glioblastoma. U87MG EGFRvIII was inoculated subcutaneously into the flank of immunodeficient NOG-ΔMHC mice on day 0, followed by intravenous administration with Conv. CAR-T, 7 × 19 CAR-T, or UTD on day 10. U87MG EGFRvIII was completely rejected in all the mice treated with 7 × 19 CAR-T (Fig. 2A). In contrast, tumors were rejected in 3 of 13 mice but recurred in 1 mouse in those treated with Conv. CAR-T, and no tumor shrinkage was observed when treated with UTD. The tumor-suppressive effect of 7 × 19 CAR-T was also observed when the mice were treated with a lower number of 7 × 19 CAR-T, i.e., 3 × 10^6^ cells, although its effect was less potent compared with that with 1 × 10^7^ cells (Supplementary Fig. S2), suggesting that a potency of 7 × 19 CAR-T is at least three times higher than that of Conv. CAR-T. The survival of tumor-inoculated mice was significantly prolonged by the treatment with 7 × 19 CAR-T, compared with that with Conv. CAR-T (Fig. 2B). These results indicated that 7 × 19 CAR-T induced potent therapeutic effects in the xenograft solid tumor model of human glioblastoma, consistent with our previous findings of 7 × 19 CAR-T in other solid tumor models (Fig. 2; refs. 20–22).

### Enhanced T-cell infiltration and tumor cell death in the tumor tissues by the treatment with anti-EGFRvIII 7 × 19 CAR-T

**Figure 3:**
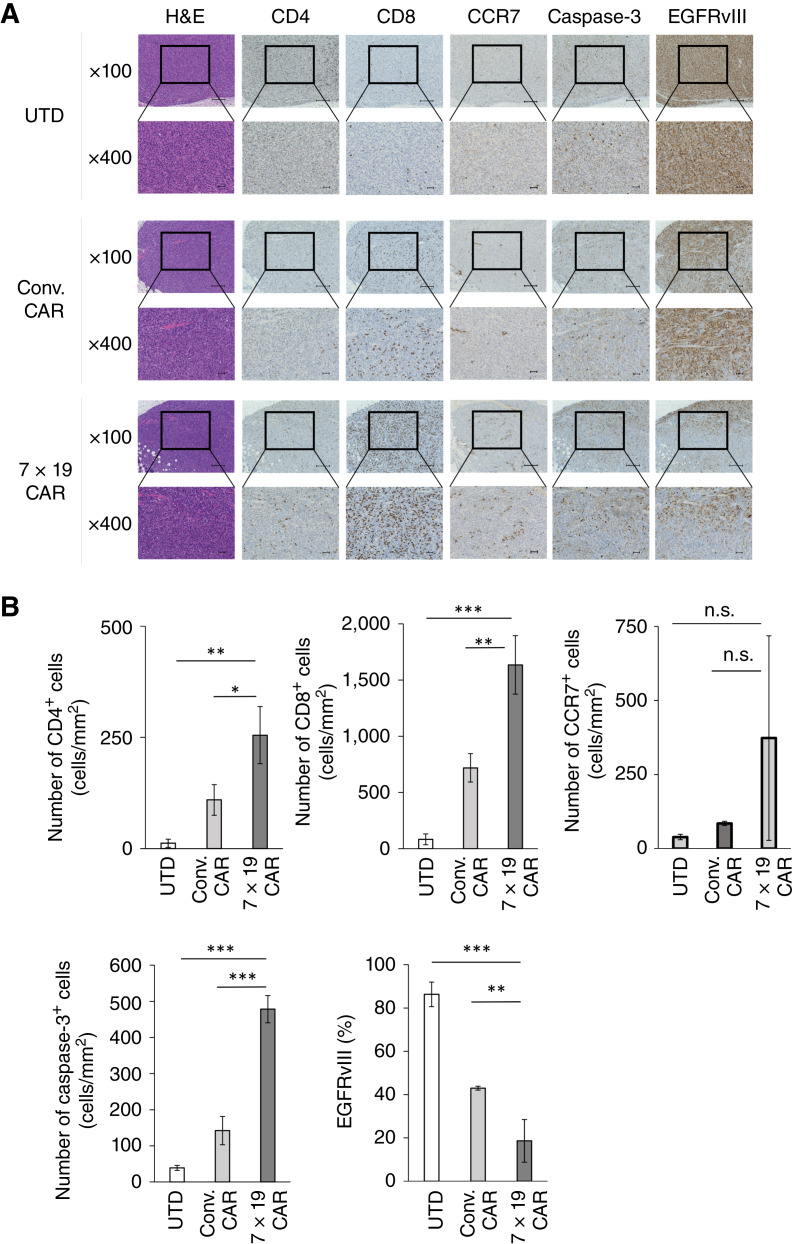
Massive infiltration of T cells and tumor cell lysis in the tumor tissues by the treatment with 7 × 19 CAR‐T. NOG‐ΔMHC mice were inoculated subcutaneously with 3.5 × 10^6^ U87MG EGFRvIII cells on day 0, followed by intravenous injection of 1 × 10^7^ UTD, Conv. CAR‐T, or 7 × 19 CAR‐T cells on day 10. Tumor tissues were resected from the mice on day 13 to prepare formalin-fixed, paraffin-embedded slices for H&E and IHC staining using rabbit anti–human CD4, CD8, CCR7, caspase-3 mAbs and mouse anti–human EGFRvIII mAb. Stained cells were visualized and observed by microscopic examinations at ×100 and ×400 magnifications. **A,** Representative images are displayed. Scale bar indicates a length of 200 μm (×100) or 50 μm (×400). **B,** The number of stained cells per tumor area (mm^2^) or the percentage of the stained area was calculated using BZ‐X analyzer software. Data are shown as mean ± SD of triplicate samples (*n* = 3 each per group, biological replicates). *, *P* < 0.05; **, *P* < 0.01; ***, *P* < 0.001; n.s., not significant.

To elucidate the immunologic mechanisms of 7 × 19 CAR-T, tumor tissues were excised from the mice that were treated with 7 × 19 CAR-T, Conv. CAR-T, or UTD and analyzed for T-cell infiltration, tumor cell death, and expression of EGFRvIII by IHC staining. Massive infiltration of T cells, especially CD8-positive T cells, was observed in the tumor tissues of the mice treated with 7 × 19 CAR-T, more aggressively than those by Conv. CAR-T treatment (Fig. 3A and B). In addition, a number of CCR7-positive SCM and central memory T cells tended to increase in the tumor tissues of the mice treated with 7 × 19 CAR-T, although it did not reach a statistical significance. Tumor lysis detected by caspase-3 expression was induced only 3 days after the treatment with 7 × 19 CAR-T. In addition, the percentage of EGFRvIII-positive cells in the tumor tissue was significantly decreased by the treatment with 7 × 19 CAR-T compared with Conv. CAR-T. These results indicated that 7 × 19 CAR-T treatment quickly induced massive T-cell infiltration and elimination of EGFRvIII-positive glioblastoma in tumor tissue (Fig. 3).

To further address the antitumor mechanisms of 7 × 19 CAR-T, its cellular phenotype was analyzed. Compared with Conv. CAR-T, 7 × 19 CAR-T, especially CD4-positive ones, contained a higher percentage of SCM phenotypes before the injection into the mouse (Supplementary Fig. S3). This distinct phenotype was maintained *in vivo*, even after the injection into the mouse, suggesting that IL7 and/or CCL19 kept converting CAR-T cells to SCM phenotypes, which is known to be a more effective cellular source for adoptive immunotherapy (29, 30). Taken together with the increase in CCR7-positive T cells in tumor tissues (Fig. 3), the induction of SCM phenotypes could be one of the potential mechanisms of the enhanced antitumor efficacy of 7 × 19 CAR-T.

### Analysis of T-cell clonality in the mice treated with 7 × 19 CAR-T

**Figure 4:**
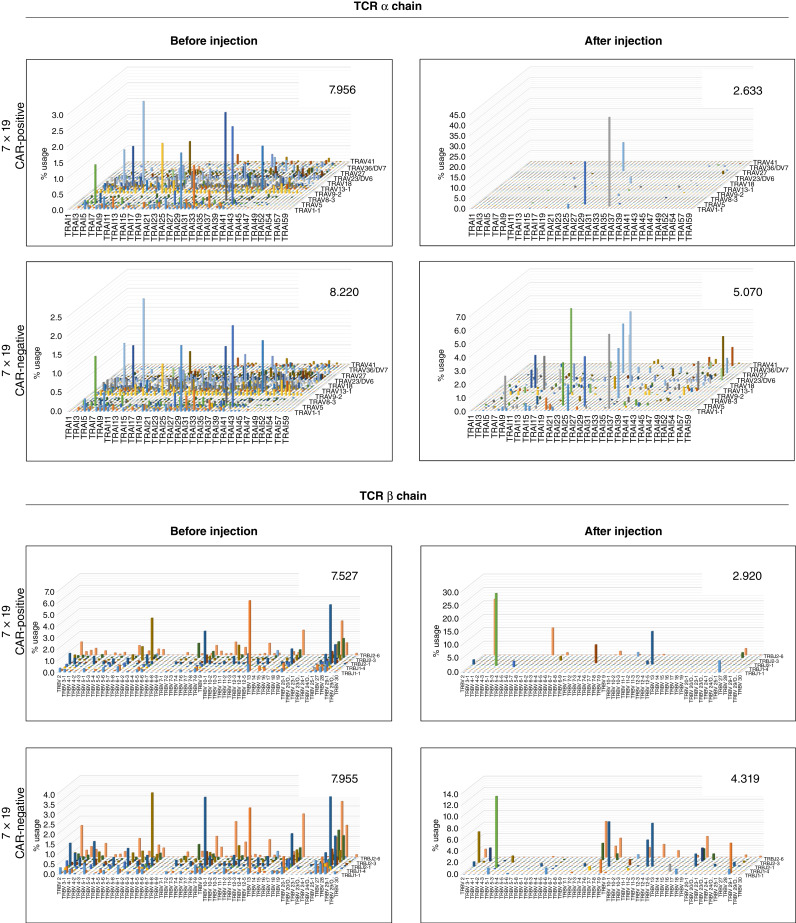
The diversity of TCR repertoire in T cells before and after the injection of 7 × 19 CAR-T. 7 × 19 CAR-T cells generated from PBMC were sorted into CAR-positive or -negative T cells and analyzed for repertoire of TCRα and TCRβ chains (before injection). Spleen cells were harvested from the mice which rejected U87MG EGFRvIII by the injection of anti-EGFRvIII 7 × 19 CAR, 42 days after tumor inoculation. Spleen cells were sorted into CAR-positive or -negative T cells for analysis of TCRα- and TCRβ-chain repertoire (after injection). TCR repertoire was analyzed using the next-generation sequencer, and the frequencies of V and J region usage were displayed as 3D graphs. The numbers in the graphs represent the diversity index calculated as Shannon–Weaver index H (a lower number indicates a less diversity). Representative data from three samples per group are shown.

To further analyze how 7 × 19 CAR-T cells demonstrate its efficacy, we next explored the change in T-cell clonality of CAR-T and non–CAR-T cells before and after injection. First, anti-EGFRvIII 7 × 19 CAR-T cells generated from PBMC were sorted into CAR-positive and -negative populations and subjected to TCR repertoire analysis to obtain the data before injection. Next, spleen cells were harvested from the mice which rejected the U87MG EGFRvIII by the injection of anti-EGFRvIII 7 × 19 CAR-T, and sorted into CAR-positive and -negative T cells for TCR repertoire analysis to obtain the data after injection. Diversity of TCRα- and TCRβ-chain repertoire in CAR-positive T cells was remarkably decreased after injection, compared with those before injection, resulting in almost oligoclonal populations (Fig. 4). In addition, the diversity of TCRα- and TCRβ-chain repertoire was also decreased in CAR-negative T cells after injection, although not as skewed as that of CAR-positive T cells. These findings suggested that limited numbers of clones in 7 × 19 CAR-T could activate, proliferate, and become memory T cells to mediate antitumor responses and that immune responses of non–CAR-T cells were also modified by the effects of 7 × 19 CAR-T (Fig. 4).

### *In vitro* antitumor responses of patient’s PBMC-derived anti-HER2 CAR-T cells against autologous pancreatic cancer organoids

**Figure 5:**
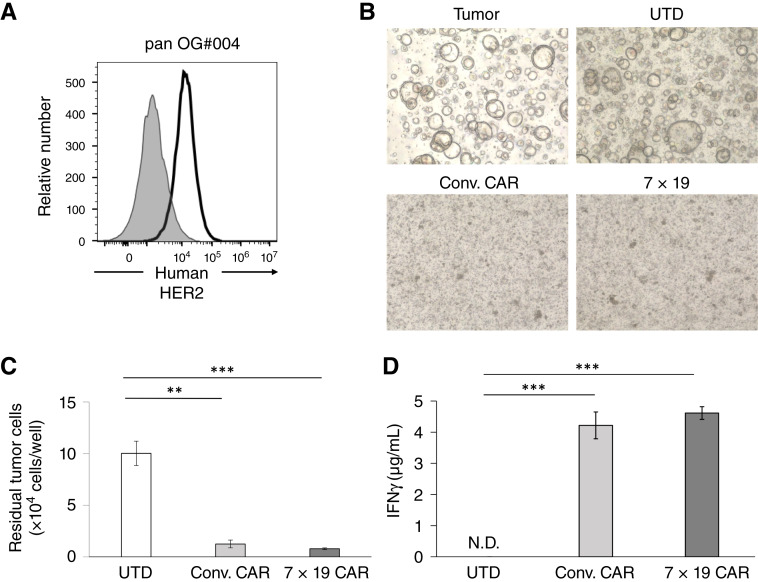
*In vitro* responses of autologous Conv. and 7 × 19 CAR-T against patient-derived pancreatic cancer organoids. **A,** Surface expression of endogenous HER2 was assessed in patient-derived pancreatic cancer organoids by flow cytometry. Open and filled histograms indicate staining with humanized anti-HER2 Ab and nonstaining, followed by anti–human IgG Ab, respectively. **B–D,** Anti-HER2 Conv. or 7 × 19 CAR-T or UTD was cocultured with HER2-positive pancreatic cancer organoids at an effector to target (E:T) ratio of 1:1 for 4 days. **B,** Morphologic images after 4 days of coculture. **C,** The number of residual tumor cells after coculture was analyzed by flow cytometry. Data are shown as mean ± SD of triplicate samples. **D,** The supernatants were harvested after 4 days of coculture, and the concentration of IFNγ was assessed by ELISA. Data are shown as mean ± SD of triplicate samples. **, *P* < 0.01; ***, *P* < 0.001; N.D., not detected.

In previous experiments, therapeutic effects and mechanisms of CAR-T were examined in mouse models using healthy donor–derived PBMC and allogeneic tumor cell lines, which were generated under conditions different from actual clinical situations. For more accurate evaluation in a model with a clinical similarity, we next attempted to examine 7 × 19 CAR-T cells generated from PBMC derived from patients with cancer for their effects against autologous cancer cells. To this end, we first established patient-derived organoids of pancreatic cancer from the specimens resected by operation and screened them by flow cytometry to detect antigens for CAR targets. Pancreatic cancer organoids with HER2 expression on the cell surface were identified (Fig. 5A) and used for inoculation into NOG-ΔMHC mice. We also generated anti-HER2 Conv. or 7 × 19 CAR-T from PBMC of the autologous patient (Supplementary Fig. S4A–S4C). When HER2-positive pancreatic cancer organoids were cocultured with Conv. or 7 × 19 CAR-T, the number of residual tumor cells was significantly reduced compared with UTD (Fig. 5B and C). In addition, IFNγ production by Conv. and 7 × 19 CAR-T, but not UTD, was detected in the coculture with HER2-positive pancreatic cancer organoids (Fig. 5D). The number of residual tumors and the level of IFNγ production were comparable between Conv. and 7 × 19 CAR-T. These results confirmed that CAR-T generated from patient’s PBMC induced antitumor responses against autologous pancreatic cancer organoids *in vitro* (Fig. 5).

### Potent therapeutic effects of 7 × 19 CAR-T generated from patient’s PBMC against autologous pancreatic cancer organoids *in vivo*

**Figure 6:**
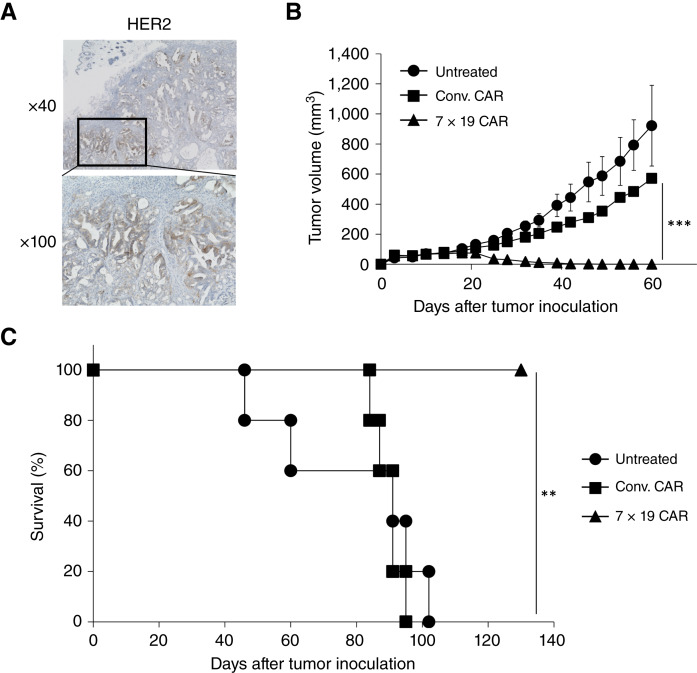
Potent therapeutic effects of patient’s PBMC-derived 7 × 19 CAR-T in autologous pancreatic cancer organoids. **A,** Tumor mass was established in NOG-ΔMHC by subcutaneous inoculation with 1 × 10^6^ pancreatic cancer organoids. After 88 days, the established tumor mass was resected and examined for HER2 expression by IHC staining. **B** and **C,** NOG-ΔMHC mice were inoculated subcutaneously with 1 × 10^6^ pancreatic cancer organoids on day 0 and then treated with intravenous injection of 5 × 10^6^ anti-HER2 Conv. CAR-T or 7 × 19 CAR-T cells on day 10 or (left) untreated. Thereafter, the tumor size (**B**) and the mouse survival (**C**) were assessed (*n* = 5 each in all groups, biological replicates). **, *P* < 0.01; ***, *P* < 0.001.

We next investigated the therapeutic efficacy of anti-HER2 CAR-T generated from patient’s PBMC against autologous HER2-positive pancreatic cancer organoids *in vivo*. The expression of HER2 in the solid tumor mass established by subcutaneous inoculation of pancreatic cancer organoids in NOG-ΔMHC mice was confirmed by IHC staining (Fig. 6A). The expression pattern of HER2 on the glandular epithelium in the established tumor mass was matched to that in the surgically resected specimens used for organoid generation (Supplementary Fig. S4D).

NOG-ΔMHC mice inoculated subcutaneously with HER2-positive pancreatic cancer organoids were treated intravenously with anti-HER2 Conv. or 7 × 19 CAR-T cells, or left untreated. The established tumor mass was completely rejected by the administration of 7 × 19 CAR-T (Fig. 6B), whereas Conv. CAR-T administration showed only a slight tumor shrinkage compared with the untreated group. Consistently, the mice survival was significantly extended by the treatment with 7 × 19 CAR-T, but not Conv. CAR-T, compared with the untreated group (Fig. 6C). These results indicated that 7 × 19 CAR-T cells generated from PBMC of patients with pancreatic cancer induced potent therapeutic effects against autologous pancreatic cancer established *in vivo* (Fig. 6).

## Discussion

In this study, we revealed potent therapeutic effects of 7 × 19 CAR-T cells against EGFRvIII-positive glioblastoma and HER2-positive pancreatic cancer in animal models. This effect was associated with the increased proportion of SCM phenotypes and the skewed TCR repertoire in CAR-T cells along with a massive infiltration of immune cells in the tumor tissues. Our results suggest that 7 × 19 CAR-T could be a novel therapeutic option for glioblastoma and pancreatic cancer, which are ICI-resistant, intractable solid cancers with very poor prognosis.

Our previous studies revealed that 7 × 19 CAR-T cells induce massive infiltration of immune cells, including CAR-negative T cells, in the tumor tissues and long-term memory responses, leading to significant enhancement of therapeutic efficacy against solid tumors (20–22). In this study, we found that 7 × 19 CAR-T therapy induced T-cell infiltration in the tumor site and tumor cell death as early as 3 days after administration into mice. Although caspase-3 is an early apoptosis marker, the area with positive staining of caspase-3 was not necessarily consistent with the area negative for EGFRvIII. In addition, at this point, tissue changes indicative of tumor death, such as postinflammatory fibrosis, were not found in H&E staining. Based on these results, the decrease in EGFRvIII-positive cells might not be directly connected with cell death, and thus a possibility of antigen loss or downregulation for the reason of our findings cannot be ruled out. It was also found that 7 × 19 CAR-T showed a relative increase in SCM phenotypes, compared with Conv. CAR-T, and maintained such features even after *in vivo* administration. In this regard, the function of IL7 to generate less differentiated T cells with a stem-like phenotype and self-renewal ability, especially in CD4-positive T cells, has been indicated (31, 32). In addition, less differentiated T cells with SCM phenotypes have been reported as more effective cellular sources for adoptive immunotherapy (29, 30). Our findings in the current study are consistent with these previous reports, highlighting an importance of IL7 produced by 7 × 19 CAR-T and the phenotypic changes in T cells in response to IL7 for the enhanced antitumor efficacy. In this regard, it is of interest that IFNγ produced by 7 × 19 CAR-T was relatively higher than that produced by Conv. CAR-T (Fig. 1D). As a potential mechanism for this finding, 7 × 19 CAR-T induced by our culture condition showed a unique feature with a simultaneous increase in SCM and effector phenotypes (Supplementary Fig. S3).

Our previous study using mouse CAR-T demonstrated that administration of 7 × 19 CAR-T induced the skewing of TCR repertoire in both CAR-positive and -negative T cells in the tumor-rejected mice (20). In the current study, reproducibility of this phenomenon was confirmed in human PBMC–derived 7 × 19 CAR-T. In terms of CAR-positive T cells, our findings suggest that only the limited clones, but not all, of the administered CAR-T cells undergo activation and proliferation to exert antitumor responses and become long-term memory T cells. This idea aligns with the findings in a recent study about the CAR-T clinical trial with a 10-year follow-up, in which skewed TCR repertoire of the residual CAR-T cells was observed in long-term survived patients (33). In terms of CAR-negative T cells, we previously reported an increased infiltration of CAR-negative T cells in the tumor tissues of 7 × 19 CAR-T-treated mice (21, 22). Together with the current result showing a skewed TCR clonality, it is suggested that IL7 and CCL19 produced by 7 × 19 CAR-T induce accumulation and proliferation of CAR-negative T cells expressing limited TCR with a reactivity to tumor antigens. At present, however, other possibilities including (i) xenogeneic responses of injected human T cells against mouse tissues and (ii) homeostatic proliferation of the injected T cells could not be excluded as mechanisms of the skewed TCR repertoire of CAR-negative T cells.

Due to much higher availability of experimental materials, healthy donor–derived PBMC and established tumor cell lines are generally used for the models to evaluate CAR-T functions and therapeutic potentials. In such models, however, allogeneic responses of T cells against tumor cells due to mismatch of MHC could generate unwanted effects in addition to antitumor responses by CAR-T cells. In addition, healthy donor–derived CAR-T cells could generate more robust responses compared with those from patients with cancer, whose immune systems are damaged by exposures to anticancer treatments including chemotherapeutic drugs and irradiation, thus possibly inducing an overestimated effectiveness when healthy donor–derived CAR-T cells are used. To address these issues, we evaluated 7 × 19 CAR-T in autologous transplantation models using patient-derived pancreatic cancer organoids established from surgically resected specimens and CAR-T cells generated from the same patient’s PBMC. In the current study using such an autologous model, 7 × 19 CAR-T exhibited superior antitumor effects over Conv. CAR-T, suggesting a *bona fide* effectiveness of 7 × 19 CAR technology. To the best of our knowledge, no previous publications have demonstrated the antitumor effects of armored CAR-T in autologous models of solid cancers. This autologous model would also be useful as a screening system to predict the efficacy of CAR-T cells generated from patients’ PBMC against their own tumor cell–derived organoids, which would be an important selection process of the optimal therapy for patients with cancer.

Although our current study exploited the model using subcutaneous injection of EGFRvIII-positive glioblastoma, several recent studies reported orthotopic models of glioblastoma in which mice receive an intracranial injection of tumor (34–36). In these orthotopic models, intracranial injection of CAR-T demonstrated antitumor effects superior to intravenous injection while armoring the CAR-T to produce IL15 and the combination with intratumor delivery of IL12 enhanced therapeutic efficacy of CAR-T injected intravenously in orthotopic models. The therapeutic potential and underlying mechanisms of 7 × 19 CAR-T treatment, either intravenously or intracranially, in orthotopic models of EGFRvIII-positive glioblastoma will be investigated and presented in our future reports.

In summary, this study revealed that the IL7/CCL19 production technology confers potent antitumor efficacy on CAR-T targeting EGFRvIII or HER2 and can be applicable to CAR-T against intractable solid cancers such as glioblastoma and pancreatic cancer. The effectiveness of 7 × 19 CAR-T was confirmed in an autologous transplant model using the patient’s own tumor and PBMC for the generation of organoids and CAR-T, respectively. Taken together, 7 × 19 CAR-T could be an innovative and promising therapeutic option for the treatment of intractable solid cancers.

## Supplementary Material

Supplementary Figure 1 Supplementary Figure 1. Generation and characterization of anti-EGFRvⅢ CAR-T cells expressing IL-7 and CCL19.

Supplementary Figure 2 Supplementary Figure 2. Therapeutic effects of lower number of 7×19 CAR-T in pre-established solid tumor model of human glioblastoma.

Supplementary Figure 3 Supplementary Figure 3. Immunophenotypic analyses of 7×19 CAR-T.

Supplementary Figure 4 Supplementary Figure 4. Generation and characterization of anti-HER2 CAR-T cells expressing IL-7 and CCL19.
