# Supplementary material for: Therapeutic Efficacy of IL7/CCL19-Expressing CAR-T Cells in Intractable Solid Tumor Models of Glioblastoma and Pancreatic Cancer
Source: Cancer Res Commun. 2024 Sep 25;4(9):2514–24. doi: 10.1158/2767-9764.CRC-24-0226 (PMC11423281; doi:10.1158/2767-9764.CRC-24-0226)
Supplement: Supplementary Figure 1 — Generation and characterization of anti-EGFRvⅢ CAR-T cells expressing IL-7 and CCL19. [file crc-24-0226_supplementary_figure_1_suppsf1.pdf]

Supplementary Figure 1

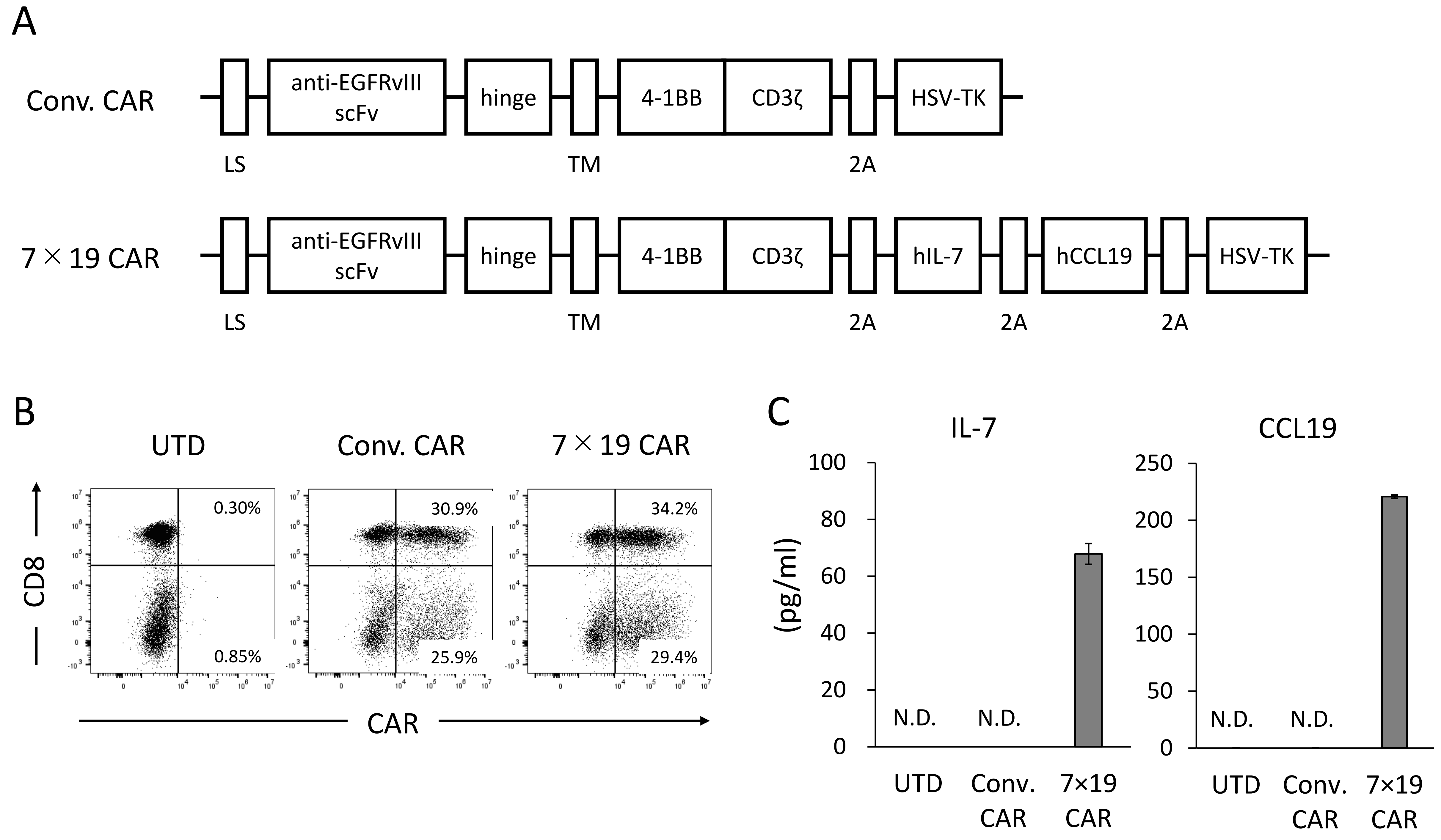

Supplementary Figure 1. Generation and characterization of anti-EGFRvⅢ CAR-T cells expressing IL-7 and CCL19. (A) Schematic representation of Conv. CAR and 7×19 CAR against EGFRvⅢ is shown. LS; leader sequence, TM; transmembrane region, 2A; self-cleavable 2A linker. (B) Human PBMC transduced with Conv. CAR or 7×19 CAR were stained with anti-CAR linker Ab to detect CAR expression, along with anti-CD8 Ab. UTD were examined as a negative control. The percentage of cells in each quadrant are indicated. (C) The culture supernatants from Conv. CAR-T and 7×19 CAR-T cells were harvested 4 days after gene transduction, and the concentrations of IL-7 and CCL19 were measured by ELISA. As a control, the culture supernatant obtained from UTD at the same time point were examined. Data are shown as mean ± standard deviation (SD) of triplicate samples. N.D.; not detected.
