# Supplementary material for: Therapeutic Efficacy of IL7/CCL19-Expressing CAR-T Cells in Intractable Solid Tumor Models of Glioblastoma and Pancreatic Cancer
Source: Cancer Res Commun. 2024 Sep 25;4(9):2514–24. doi: 10.1158/2767-9764.CRC-24-0226 (PMC11423281; doi:10.1158/2767-9764.CRC-24-0226)
Supplement: Supplementary Figure 2 — Therapeutic effects of lower number of 7×19 CAR-T in pre-established solid tumor model of human glioblastoma. [file crc-24-0226_supplementary_figure_2_suppsf2.pdf]

Supplementary Figure 2

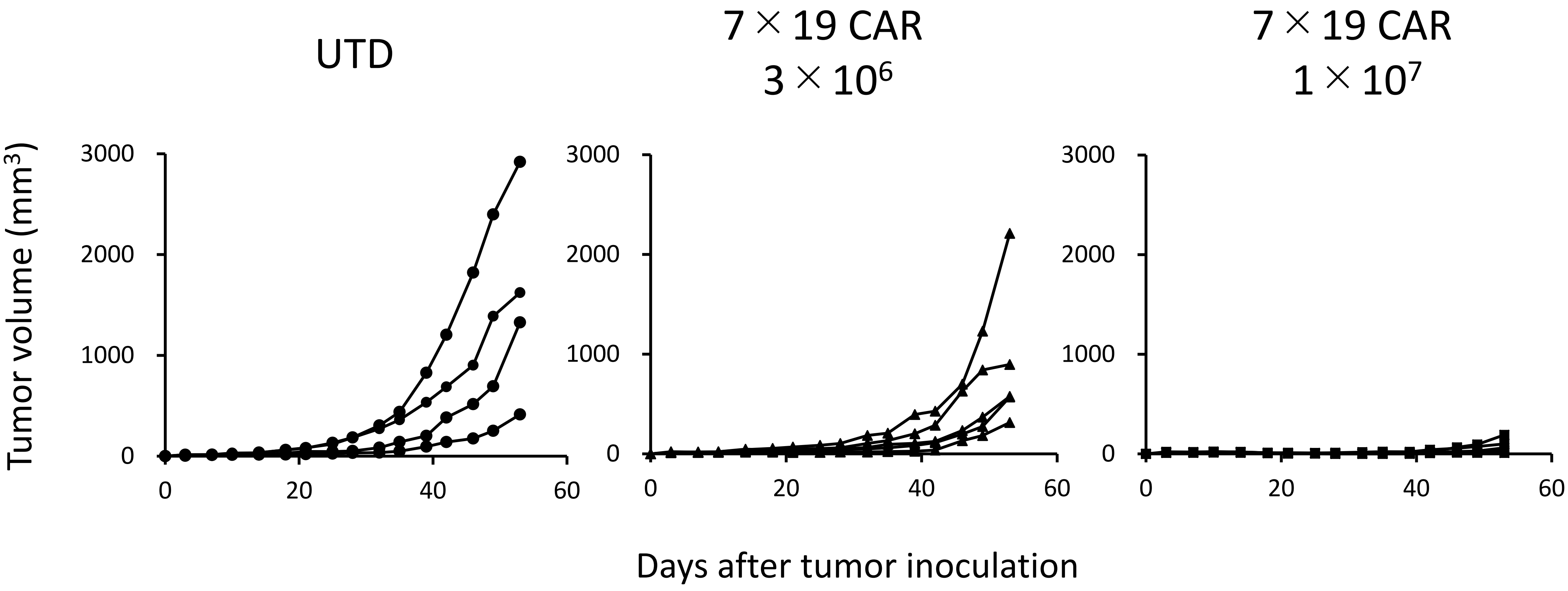

Supplementary Figure 2. Therapeutic effects of lower number of 7×19 CAR-T in pre-established solid tumor model of human glioblastoma.

NOG-ΔMHC mice were inoculated subcutaneously (s.c.) with 3.5×10^5 U87MG EGFRvⅢ tumor cells on day 0, and then treated with intravenous (i.v.) injection of UTD, 3×10^6, or 1×10^7 7×19 CAR-T on day 10. Thereafter, the tumor size was assessed twice per week (n=5 each in 7×19 CAR-T groups, n=4 in UTD group, n values are biological replicates). Each line indicates the tumor volume of individual mouse.
