# Supplementary material for: Therapeutic Efficacy of IL7/CCL19-Expressing CAR-T Cells in Intractable Solid Tumor Models of Glioblastoma and Pancreatic Cancer
Source: Cancer Res Commun. 2024 Sep 25;4(9):2514–24. doi: 10.1158/2767-9764.CRC-24-0226 (PMC11423281; doi:10.1158/2767-9764.CRC-24-0226)
Supplement: Supplementary Figure 3 — Immunophenotypic analyses of 7×19 CAR-T. [file crc-24-0226_supplementary_figure_3_suppsf3.pdf]

Supplementary Figure 3

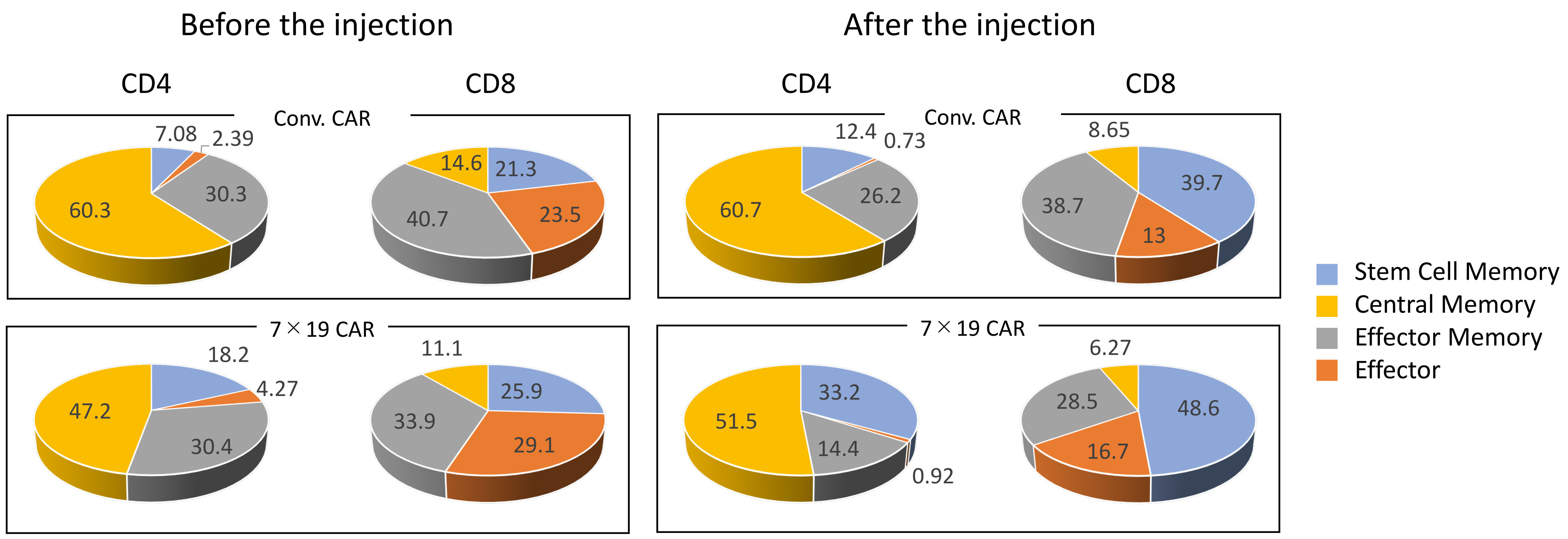

Supplementary Figure 3. Immunophenotypic analyses of 7×19 CAR-T.

Before injection into the mice, Conv. or 7×19 CAR-T were analyzed by flow cytometry to determine the percentage of stem cell memory, central memory, effector memory, and effector phenotypes, along with CD4 and CD8 populations. For *in vivo* experiments, the mice were inoculated s.c. with U87MG EGFRvⅢ cells on day 0, followed by i.v. injection of Conv. CAR-T or 7×19 CAR-T cells on day 10. Spleen were harvested from the mice on day 13, and analyzed for phenotypes of CAR-T cells as above. Immunophenotype were defined as follows; stem cell memory: $CD45RA^{+}CCR7^{+}$, central memory: $CD45RA^{-}CCR7^{+}$, effector memory: $CD45RA^{-}CCR7^{-}$, effector: $CD45RA^{+}CCR7^{-}$.
